# Appropriate pre-transplant strategy for patients with myelodysplastic syndromes receiving allogeneic haematopoietic stem cell transplantation after myeloablative conditioning

**DOI:** 10.3389/fimmu.2023.1146619

**Published:** 2023-02-28

**Authors:** Hong Wang, Qingyuan Wang, Jiaqian Qi, Xueqian Li, Tiantian Chu, Huiying Qiu, Chengcheng Fu, Xiaowen Tang, Changgeng Ruan, Depei Wu, Yue Han

**Affiliations:** ^1^ National Clinical Research Centre for Haematologic Diseases, Jiangsu Institute of Haematology, The First Affiliated Hospital of Soochow University, Suzhou, China; ^2^ Institute of Blood and Marrow Transplantation, Collaborative Innovation Centre of Haematology, Soochow University, Suzhou, China; ^3^ Key Laboratory of Thrombosis and Haemostasis of Ministry of Health, Suzhou, China; ^4^ State Key Laboratory of Radiation Medicine and Protection, Soochow University, Suzhou, China

**Keywords:** myelodysplastic syndrome, pre-transplant strategy, myeloablative conditioning, haematopoietic stem cell transplantation, prognosis

## Abstract

**Purpose:**

Appropriate pre-transplant strategies in patients with myelodysplastic syndromes (MDS) remain challenging. We sought to assess the effect of different pre-transplant therapies and transplantation interval times on patient prognosis.

**Methods:**

We retrospectively analysed clinical data for 371 consecutive MDS patients after myeloablative transplantation between 2007 and 2019.

**Results:**

The median age of the patients was 38 years (range, 12–64 years). A total of 114 patients (31%) received supportive care (SC), 108 (29%) hypomethylating agents (HMAs), and 149 (40%) chemotherapy-based therapy before transplantation. In patients who received HMA or SC, there was no significant difference in overall survival (OS; P=0.151) or relapse-free survival (RFS; P=0.330), except that HMA-treated patients had a lower rate of non-relapse mortality (5-year NRM: 18% vs. 32%, P=0.035). However, compared with patients who received HMA, those who received chemotherapy-based therapy had a lower 5-year OS rate (56% vs. 69%, P=0.020) and a slightly higher 5-year NRM rate (28% vs. 18%, P=0.067). Compared to the delayed transplant group (transplant interval ≥6 months), the early transplant group (transplant interval <6 months) had a superior 5-year OS (66% vs. 51%, P=0.001) and a lower 5-year cumulative incidence of NRM (22% vs. 36%, P=0.001).

**Conclusion:**

The findings of the study indicate that receiving an appropriate pre-transplant strategy (SC/HMA + <6 months) significantly improves OS and decreases NRM in MDS patients after myeloablative transplantation.

## Introduction

Myelodysplastic syndromes (MDS) are a group of clonal haematopoietic stem cell disorders characterized by cytopaenia, ineffective haematopoiesis, and increased risk of evolution to acute myeloblastic leukaemia (AML) (1). With the growing understanding of the pathophysiology of MDS, epigenetic therapy with hypomethylating agents (HMAs), including azacitidine (AZA) and decitabine (DAC), has become the standard treatment. Despite their low toxicity and ability to induce a haematological response and survival improvement (2–4), HMAs are not curative therapies for MDS.

Allogeneic haematopoietic stem cell transplantation (HSCT) is the only potentially curative therapeutic option; it significantly improves overall survival (OS) and disease-free survival, depending on prognostic features (5–7). The role of different pre-transplant therapies in patients who are transplant candidates has been investigated. In a series of small retrospective analyses, HMAs were found to confer no clear benefit when given pre-HSCT (8–12). Additionally, one study comparing outcomes after chemotherapy, HMAs, or best supportive care (SC) pre-allo-HSCT reported no prognostic advantage with any treatment (13). However, sequential treatment with AZA followed or preceded by chemotherapy was found to adversely influence both OS and event-free survival (9).

In addition to pre-transplant therapies, optimal timing of allo-HSCT for a given patient with MDS is important. The most cited Markov decision model compares life expectancy after myeloablative conditioning (MAC) transplantation for younger patients, showing that early transplantation is associated with maximal life expectancy in patients with International Prognostic Scoring System (IPSS) intermediate-2 and high-risk disease stages (14). Koreth et al. employed the Markov decision model and found similar results for older patients who received HSCT in the reduced intensity conditioning (RIC) setting (15). As different pre-transplant therapies result in different interval times between diagnosis and transplantation, the decision of an appropriate pre-transplantation strategy presents many challenges when considering these two factors.

The aim of this study was to assess the effect of different pre-transplant therapies (HMA, chemotherapy and SC) and transplantation interval times on patient prognosis. Based on the results, we propose an appropriate pre-transplant strategy for MDS patients who received allo-HSCT after MAC.

## Patients and methods

### Patient selection

Between December 2007 and September 2019, 415 consecutive MDS patients underwent allo-HSCT at the First Affiliated Hospital of Soochow University. To exclude the impact of different conditioning approaches, 44 patients who received the RIC conditioning regimen were excluded from our analysis. Thus, 371 patients who received allo-HSCT after the MAC conditioning regimen were included in the final analysis. Informed consent was obtained from all patients prior to data collection. This study was approved by the Committee for the Ethical Review of Research at The First Affiliated Hospital of Soochow University and conducted in accordance with institutional guidelines and the Declaration of Helsinki.

Diagnosis of MDS was based on the 2016 World Health Organization (WHO) classification (16). IPSS and revised IPSS (IPSS-R) scores were used for risk evaluation (17, 18). Patients were divided into three groups according to treatment received prior to transplantation, as follows: SC, HMA alone and chemotherapy-based treatment (chemotherapy). SC includes growth factors (erythropoietin, granulocyte colony-stimulating factor), hormones, blood transfusion, immunosuppressive treatment and antibiotics. HMA includes DAC (20 mg/m^2^ on Days 1–5, for each cycle) or AZA (75 mg/m^2^ on Days 1–7, for each cycle). Chemotherapy-based treatment includes induction chemotherapy alone and HMA plus chemotherapy. Induction chemotherapy includes low-dose CAG or revised CAG regimens (IAG and HAG). The CAG regimen consisted of cytarabine at 10 mg/m^2^, q12 h on Days 1–7; aclarubicin at 7 mg/m^2^, qd on Days 1–4; and G-CSF at 200 μg/m^2^, qd on Days 1–7. The IAG regimen consisted of idarubicin at 8 mg/m^2^, qd on Days 1–3; cytarabine at 10 mg/m^2^, q12 h on Days 1–7; and G-CSF at 200 μg/m^2^, qd on Days 1–7. HAG consisted of homoharringtonine at 2 mg/m², qd, on Days 1–3; cytarabine at 10 mg/m^2^, q12 h on Days 1–7; and G-CSF at 200 μg/m^2^, qd on Days 1–7. For HMA plus chemotherapy, hypomethylating agents were administered before CAG or revised CAG regimens.

### Transplantation modalities

A total of 115 patients (31%) received HLA-matched related donor transplantation (MRDT), 66 patients (18%) received HLA-matched unrelated donor transplantation (MUDT), and 190 patients (51%) received haploidentical stem cell transplantation (haplo-SCT). All patients were followed until death or June 2021. The patient characteristics are shown in **Table 1**.

**Table 1:** Patient characteristics according to different prior-to-transplant therapies.

Variables	Total (n = 371)	Supportive care (n = 114)	HMA (n = 108)	Chemotherapy (n = 149)	P value
**Sex, n (%)**					0.510
Female	141 (38)	40 (35)	39 (36)	62 (42)	
Male	230 (62)	74 (65)	69 (64)	87 (58)	
**Age, n (%)**					**< 0.001**
< 40	180 (49)	75 (66)	39 (36)	66 (44)	
≥ 40	191 (51)	39 (34)	69 (64)	83 (56)	
**WBC (**10^9^/L**), n (%)**					0.071
< 4	296 (80)	98 (86)	87 (81)	111 (74)	
≥4	75 (20)	16 (14)	21 (19)	38 (26)	
**HB (**g/L**), n (%)**					0.610
<100	295 (80)	90 (79)	83 (77)	122 (82)	
≥100	76 (20)	24 (21)	25 (23)	27 (18)	
**PLT** (10^9^/L)**, n (%)**					**0.020**
< 50	210 (57)	76 (67)	53 (49)	81 (54)	
≥ 50	161 (43)	38 (33)	55 (51)	68 (46)	
**Blast, n (%)**					**< 0.001**
< 5%	132 (36)	73 (64)	40 (37)	19 (13)	
≥ 5%	239 (64)	41 (36)	68 (63)	130 (87)	
**IPSS karyotype, n (%)**					0.152
Good	210 (57)	71 (62)	55 (51)	84 (56)	
Int	103 (28)	33 (29)	31 (29)	39 (26)	
Poor	58 (16)	10 (9)	22 (20)	26 (17)	
**WHO classification, n (%)**					**< 0.001**
EB-1	100 (27)	35 (31)	33 (31)	32 (21)	
EB-2	143 (39)	8 (7)	34 (31)	101 (68)	
Other	128 (35)	71 (62)	41 (38)	16 (11)	
**IPSS risk, n (%)**					**< 0.001**
Low	3 (1)	2 (2)	0 (0)	1 (1)	
Int-1	188 (51)	90 (79)	56 (52)	42 (28)	
Int-2	140 (38)	22 (19)	41 (38)	77 (52)	
High	40 (11)	0 (0)	11 (10)	29 (19)	
**IPSS-R risk, n (%)**					**< 0.001**
Low	18 (5)	10 (9)	6 (6)	2 (1)	
Int	99 (27)	44 (39)	31 (29)	24 (16)	
High	158 (43)	53 (46)	45 (42)	60 (40)	
Very high	96 (26)	7 (6)	26 (24)	63 (42)	
**Secondary MDS, n (%)**					**0.010**
No	311 (84)	85 (75)	95 (88)	131 (88)	
yes	60 (16)	29 (25)	13 (12)	18 (12)	
**Disease status before HSCT, n (%)**					**< 0.001**
CR/mCR	88 (24)	0 (0)	26 (24)	62 (42)	
Other	283 (76)	114 (100)	82 (76)	87 (58)	
**Disease progression before HSCT, n (%)**					0.094
No	277 (75)	92 (81)	82 (76)	103 (69)	
Yes	94 (25)	22 (19)	26 (24)	46 (31)	
**AML transformation before HSCT, n (%)**					**< 0.001**
No	343 (92)	111 (97)	106 (98)	126 (85)	
Yes	28 (8)	3 (3)	2 (2)	23 (15)	
**Interval time between diagnosis and HSCT, n (%)**					0.510
< 6 months	258 (70)	75 (66)	76 (70)	107 (72)	
6-12 months	53 (14)	14 (12)	17 (16)	22 (15)	
12-24 months	31 (8)	14 (12)	6 (6)	11 (7)	
>24 months	29 (8)	11 (10)	9 (8)	9 (6)	
**Donor type, n (%)**					0.280
Sibling donor	115 (31)	39 (34)	30 (28)	46 (31)	
Unrelated donor	66 (18)	25 (22)	15 (14)	26 (17)	
Haploidentical donor	190 (51)	50 (44)	63 (58)	77 (52)	
**Source of stem cell, n (%)**					0.090
BM	30 (8)	8 (7)	6 (6)	16 (11)	
BM+PB	201 (54)	54 (47)	68 (63)	79 (53)	
PB	140 (38)	52 (46)	34 (31)	54 (36)	

HMA, hypomethylating agents; IPSS, International prognostic scoring system; IPSS-R, revised IPSS; CR, complete remission; mCR, complete remission in morphology

Significant p values (p < 0.05) are bolded.

All patients received MAC regimens. For MRDT, the MAC regimens comprised semustine (250 mg/m^2^, day −10), cytarabine (2 g/m^2^/d, days −9 to −8), busulfan (3.2 mg/kg/d, days −7 to −5), and cyclophosphamide (1.8 g/m^2^/d, days −4 to −3). For MUDT and haplo-SCT, patients received a MAC regimen identical to that for MRDT but with higher doses of cytarabine (4 g/m^2^/d, days −9 to −8). In addition, patients receiving MUDT received hydroxycarbamide (80 mg/kg, day −10). Rabbit anti-thymocyte globulin (ATG; Genzyme Polyclonals S.A.S, Lyon, France), ATG-F (Fresenius Biotech GmbH, Munich, Germany) or porcine antilymphocyte globulin (ALG; Wuhan Institute of Biological Products Co., Ltd., Wuhan, Hubei, China) was administered to patients receiving MUDT and haplo-SCT for graft-versus-host disease (GVHD) prophylaxis. The regimens were as follows: ATG 2.5 mg/kg/day for four days; ATG-F 5 mg/kg/day for four days; and ALG 15 mg/kg/day for four days. For a small number of patients who received MSDT, a lower dose of ATG (2.5 mg/kg/day, for two days) or ATG-F (5 mg/kg/day, for two days) was used.

Patients who underwent MRDT received GVHD prophylaxis consisting of cyclosporine and methotrexate. GVHD prophylaxis in patients who underwent MUDT or haplo-SCT consisted of cyclosporine, mycophenolate mofetil, and methotrexate.

### Study endpoints, definitions, and statistical analysis

Marrow CR (mCR) indicates a clinical CR with a morphologically normal marrow but persistent cytopenia (19, 20). Overall survival (OS) was defined from the time of allo-HSCT to death, regardless of cause, or the last follow-up. Relapse-free survival (RFS) was defined as the time from allo-HSCT to treatment failure (death or relapse). NRM was defined as death from any cause in the first 28 days after allo-HSCT or death without evidence of disease recurrence beyond Day 28, with relapse as a competing event. Relapse was defined as disease morphological recurrence and/or reappearance of the underlying disease. Relapse incidence was estimated by considering relapse as the event of interest and death without relapse as a competing event.

OS and RFS were computed using the Kaplan–Meier method, and the log-rank test was used for univariate comparisons. Prognostic factors with values of P ≤ 0.1 in univariate analyses were entered into a Cox proportional hazards model to determine their effects on survival. The cumulative incidence method was applied to compute the incidence of NRM and relapse in a competing risk setting, with the Gray test applied for comparisons of different groups (21). For risk factors for cumulative incidence of relapse (CIR) and NRM, factors with values of P ≤ 0.1 in univariate analyses and those with clinical significance were chosen for further evaluation in the multivariate regression analysis proposed by Fine and Gray (22). All analyses were performed using the R software package (R version 4.0.3; The R Foundation for Statistical Computing, www.r-project.org).

## Results

A total of 371 MDS patients received allo-HSCT after MAC conditioning. Of those, 114 patients (31%) received SC, 108 received HMA (29%, DAC in 104 cases and AZA in four cases), and 149 (40%) received low-dose chemotherapy, including 20 cases of chemotherapy alone and 129 DAC plus chemotherapy. The median age of the patients was 38 years (range, 12–64 years). Baseline characteristics were well balanced between the three groups, except as expected for investigator-preselected subgroups, namely, more patients with EB-2, higher blast percentage, or higher IPSS-R risk at diagnosis received chemotherapy (**Table 1**). After transplantation, 126 patients (34%) developed grade 2 to 4 acute GVHD (aGVHD), 144 (39%) developed chronic GVHD (cGVHD), and 36 (10%) had extensive cGVHD. For the whole patient population, the 3-year OS, 3-year RFS, 3-year CIR, and 3-year cumulative incidence of NRM were 66%, 86%, 10%, and 25%, respectively.

### Outcome according to prior-to-transplant treatment

**Figure 1:**
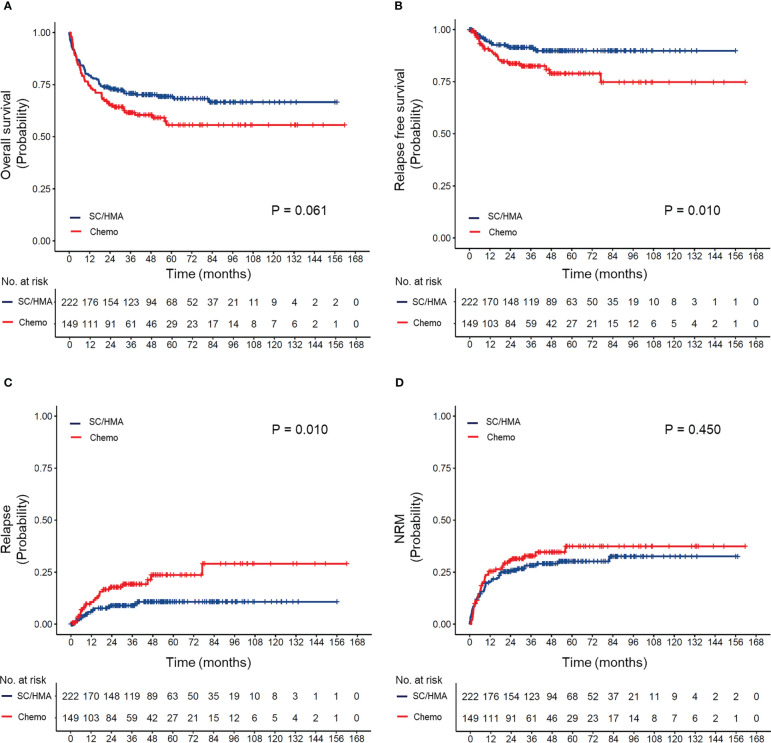
**(A)** Overall survival, **(B)** relapse-free survival, **(C)** cumulative incidence of relapse, and **(D)** cumulative incidence of non-relapse mortality (NRM) according to prior transplantation treatment received. SC, supportive care; HMA, hypomethylating agents (decitabine and azacitidine); Chemo, chemotherapy.

OS, RFS, CIR, and NRM based on different prior-to-transplant therapies are shown in **Supplemental Figure 1**. Among patients who received HMA or SC, there were no significant differences in OS (P=0.151) or RFS (P=0.330); however, patients in the HMA group had a lower rate of NRM (5-year NRM: 18% vs. 32%, P=0.035). When compared with patients who received HMA, those who received chemotherapy had lower 5-year OS rates (56% vs. 69%, P=0.020) and slightly higher 5-year NRM rates (28% vs. 18%, P=0.067). Based on these findings, we combined patients who received SC and those who received HMA into a non-chemotherapy group and found that the patients in this group had superior OS (P=0.061) and RFS (P=0.010) and a lower rate of relapse (P=0.010), with no significant difference in NRM in comparison to those in the chemotherapy group (**Figure 1**).

To a certain extent, HSCT was able to overcome the adverse prognostic impact of disease progression before transplantation, with no significant difference in terms of OS, RFS, CIR, and NRM between patients who experienced progression and those who did not. However, worse RFS and higher CIR were observed when the disease progressed to AML. The proportion of patients who achieved complete remission (CR) or marrow CR (mCR) was significantly higher in the chemotherapy group than in the HMA group (42% vs. 24%, P < 0.001). No patients in the supportive care group achieved CR or mCR. Notably, a higher response rate did not translate to a favourable prognosis in the chemotherapy group. Patients who achieved CR or mCR did not show any significant advantage in OS, RFS, CIR, or NRM (**Table 2**). Moreover, the type of treatment before HSCT had no significant effect on the incidence or severity of aGVHD or cGVHD. In addition, cytomegalovirus (CMV)/Epstein–Barr virus (EBV) reactivation did not differ among patients who received SC, HMA or chemotherapy pre-transplantation (**Table 1**).

**Table 2:** Univariate analysis of prognostic factors for OS, RFS, cumulative incidence of relapse, and cumulative incidence of NRM.

Variables	No.	5-year OS		5-year RFS		5-year CIR		5-year NRM	
		%	P #	%	P #	%	P †	%	P †
**Sex**			0.223		0.911		0.979		0.418
Male	230	59		85		11		27	
Female	141	66		82		11		24	
**Age**			**0.006**		**0.030**		**0.071**		**0.080**
< 40	180	71		87		8		22	
≥ 40	191	51		81		14		30	
**Blast**			0.272		0.224		0.177		**0.099**
< 5%	132	60		89		8		31	
≥ 5%	239	61		81		13		23	
**IPSS karyotype**			0.266		0.116		**0.040**		0.930
Good	210	63		89		8		26	
Int	103	61		77		14		27	
Poor	58	58		81		16		29	
**WHO classification**			0.216		0.601		0.467		**0.088**
EB-1	100	58		88		12		23	
EB-2	143	64		86		13		22	
Others	128	61		79		9		33	
**IPSS risk**			0.678		**0.048**		**0.092**		0.894
Lower risk	191	63		89		8		27	
Higher risk	180	59		78		41		25	
**IPSS-R risk**			0.641		0.636		0.126		0.718
Lower risk	117	59		84		12		27	
Higher risk	254	62		84		10		26	
**Secondary MDS**			0.837		0.492		0.475		0.947
No	311	61		83		12		26	
Yes	60	63		91		8		27	
**Disease status before HSCT**			0.146		0.864		0.707		0.225
CR/mCR	88	67		80		11		21	
Others	283	60		85		11		28	
**Disease progression before HSCT**			0.480		0.669		0.817		0.895
No	277	62		84		11		26	
Yes	94	62		86		12		26	
**AML transformation before HSCT**			0.202		**0.001**		**0.002**		0.496
No	343	62		85		10		27	
Yes	28	51		67		29		21	
**Prior to HSCT therapies**			**0.070**		**0.026**		**0.038**		**0.071**
Supportive care	114	62		91		6		32	
HMA	108	69		89		10		18	
Chemotherapy	149	56		74		15		28	
**Interval time between diagnosis and HSCT**			**0.009**		0.595		0.567		**0.016**
< 6 months	258	66		87		11		22	
6-12 months	53	53		81		13		32	
12-24 months	31	57		88		6		42	
> 24 months	29	39		47		14		38	
**DAC in conditioning regimen**			**0.076**		0.384		0.393		0.114
No	216	57		81		12		30	
Yes	155	68		88		10		21	
**ATG in conditioning regimen**			0.470		0.324		0.369		0.181
No	92	59		89		9		33	
Yes	279	62		82		12		24	
**Donor type**			0.234		0.940		0.806		0.302
Sibling donor	115	63		86		11		26	
Unrelated donor	66	68		78		12		20	
Haploidentical donor	190	58		86		11		28	
**Source of stem cell**			**0.010**		**0.002**		**0.009**		0.502
Not BM	341	45		85		10		25	
BM	30	62		66		27		33	
**Grade of aGVHD**			**< 0.001**		**0.008**		**0.006**		**< 0.001**
None/1	246	69		82		15		14	
2-4	126	50		89		3		45	
**Grade of cGVHD**			**< 0.001**		0.453		0.920		**< 0.001**
Extensive	36	40		84		11		57	
Others	335	70		80		11		22	
**CMV infection**			0.873		0.788		0.828		0.893
No	258	60		83		11		26	
Yes	113	65		88		11		27	
**EBV infection**			0.239		**0.001**		**< 0.001**		0.815
No	310	63		87		8		26	
Yes	61	55		68		24		26	

IPSS, International prognostic scoring system; IPSS-R, revised IPSS; CR, complete remission; mCR, complete remission in morphology; HMA, hypomethylating agents; DAC, decitabine; ATG, anti-thymocyte globulin; HLA, human leukocyte antigen; GVHD, graft-versus-host disease; aGVHD, acute GVHD; cGVHD, chronic GVHD; CMV, cytomegalovirus; EBV, Epstein-Barr virus.

Significant p values (p < 0.05) are bolded.

#Log-rank test.

†Gray test (cumulative incidence).

### Timing of transplantation

**Figure 2:**
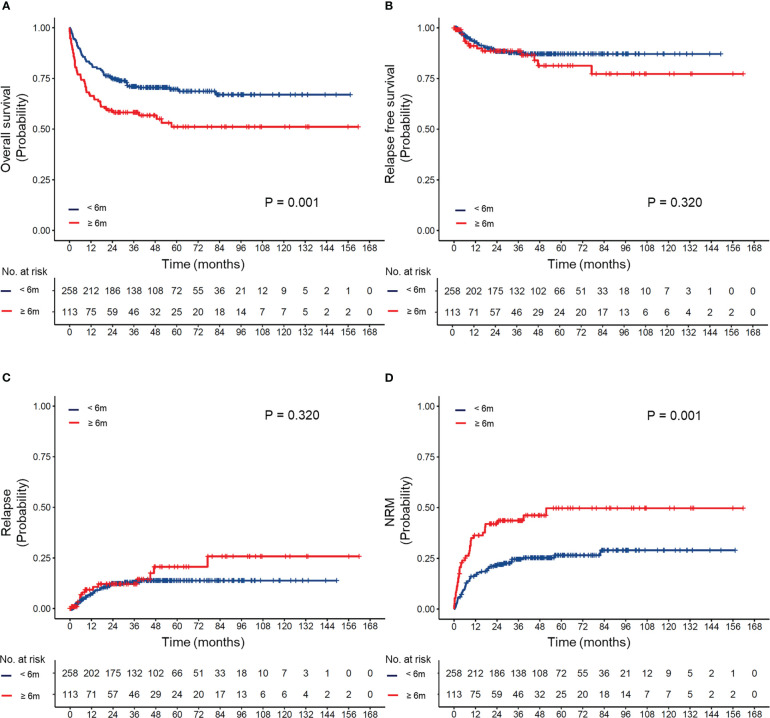
**(A)** Overall survival, **(B)** relapse-free survival, **(C)** cumulative incidence of relapse, and **(D)** cumulative incidence of non-relapse mortality (NRM) according to different intervals between diagnosis and transplantation.

To evaluate the effect of differences in the timing of transplantation on transplant outcomes, patients were divided into four groups according to the transplant interval: less than 6 months, 6–12 months, 12–24 months, and more than 24 months. The OS, RFS, CIR, and NRM of the cohort based on different transplant interval times are shown in **Supplemental Figure 2**. Compared to the delayed transplant group (transplant interval ≥6 months), the early transplant group (transplant interval <6 months) had a superior 5-year OS (66% vs. 51%, P=0.001) and a lower 5-year cumulative incidence of NRM (22% vs. 36%, P=0.001) (**Figure 2**).

**Figure 3:**
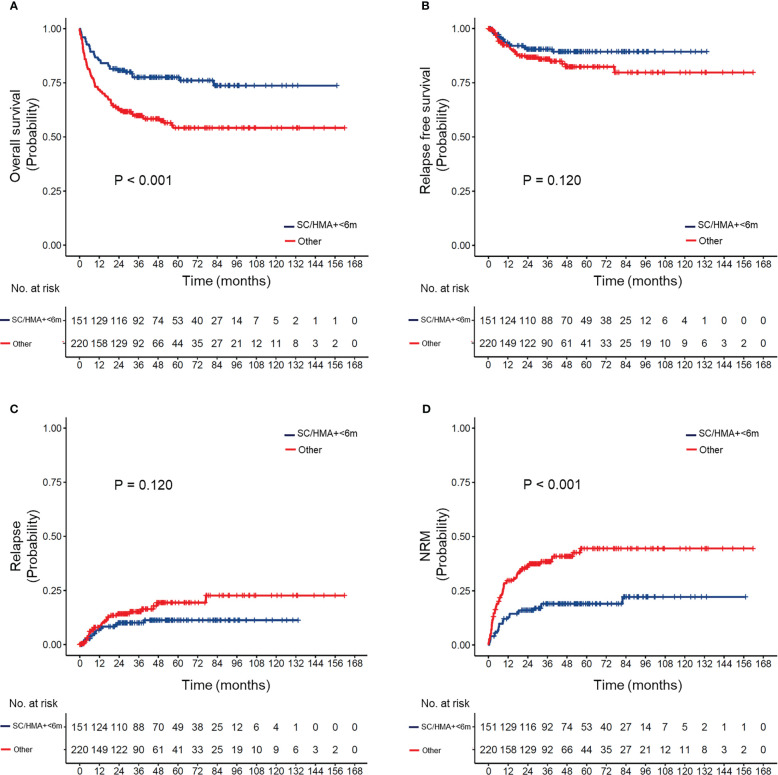
**(A)** Overall survival, **(B)** relapse-free survival, **(C)** cumulative incidence of relapse, and **(D)** non-relapse mortality (NRM) in MDS patients according to pre-transplant strategy (appropriate strategy vs. others). Appropriate strategy: MDS patients who received hypomethylating agents or supportive care and underwent HSCT within 6 months after diagnosis.

The effect of differences in the transplant interval in patients with different IPSS-R risks is provided in **Supplemental Figure 3** and **Figure 3**. Our results suggested that early transplantation conferred better OS and lower NRM after HSCT in both lower and higher-risk patients.

### Appropriate pre-transplant treatment strategy

Different pre-transplant therapies may result in different intervals between diagnosis and transplantation. Therefore, we reclassified our patients according to the type of pre-transplant therapy and timing of HSCT. According to our results, patients in the SC/HMA + <6 months group had a superior OS and a lower rate of NRM than those in the other three groups after pairwise comparison (**Supplemental Figure 4**). No significant differences in OS were observed between the SC/HMA + ≥6 months, Chemo + <6 months, and Chemo + ≥6 months groups, though patients in the Chemo + ≥6 months group showed worse RFS after transplantation (**Supplemental Figure 5**). Therefore, receiving non-chemotherapy and transplantation within 6 months after diagnosis (SC/HMA + <6 months) was regarded as the most appropriate pre-transplant strategy in MDS patients.

### Univariate analysis

Among all at-diagnosis and/or at-transplantation characteristics studied, MDS patients who aged ≥ 40 years or received marrow-derived stem cells or experienced grade 2 to 4 aGVHD had inferior OS and RFS. Appropriate treatment strategy before HSCT, grade 2 to 4 aGVHD, and extensive cGVHD were found to correlate with OS and NRM (**Table 2**). Furthermore, AML transformation before HSCT and EBV infection after HSCT were found to be associated with RFS and CIR.

### Multivariate analysis

The results of multivariate analysis of OS, RFS, CIR, and NRM are shown in **Table 3**. Older age (≥ 40 years) was an independent risk factor for OS (HR=1.55, P=0.020), RFS (HR=2.53, P=0.006), CIR (HR=2.53, P=0.005) and NRM (HR=1.63, P=0.018). Additionally, AML transformation before HSCT and EBV infection after HSCT adversely influenced RFS and CIR (**Table 3**), and grade 2 to 4 aGVHD adversely affected OS (HR=2.13, P<0.001), RFS (HR=4.01, P=0.004), CIR (HR=4.01, P=0.004), and NRM (HR=2.96, P<0.001). Moreover, the prognostic effect of an appropriate treatment strategy before HSCT on OS and NRM was confirmed in multivariate analysis (**Table 3**).

**Table 3:** Multivariate analysis for OS, RFS, CIR and NRM.

Variables	HR	95% CI	P value
Overall survival (OS)			
**Age** (≥ 40 vs. Others)	1.55	1.07-2.24	**0.020**
**Prior to HSCT therapies** (Others vs. appropriate treatment)	2.19	1.45-3.30	**< 0.001**
**DAC in conditioning** (Yes vs. No)	0.76	0.51-1.12	0.167
**Source of stem cell** (Not marrow vs. Marrow)	0.61	0.36-1.03	0.066
**Grade of aGVHD** (2-4 vs. Others)	2.13	1.47-3.13	**< 0.001**
**Grade of cGVHD** (Extensive vs. Others)	1.72	1.05-2.78	**0.032**
Relapse free survival (RFS)			
**Age** (≥ 40 vs. Others)	2.53	1.3-4.95	**0.006**
**IPSS risk** (Higher vs. Lower)	1.94	1.02-3.7	**0.045**
**AML transformation** (Yes vs. No)	3.57	1.5-8.52	**0.004**
**Prior to HSCT therapies** (Others vs. Appropriate treatment)	1.2	0.61-2.36	0.597
**Source of stem cell** (Not marrow vs. Marrow)	0.45	0.19-1.06	0.067
**Grade of aGVHD** (2-4 vs. Others)	4.01	1.56-10.29	**0.004**
**EBV infection** (Yes vs. No)	3.77	1.9-7.48	**< 0.001**
Cumulative incidence of relapse (CIR)			
**Age** (≥ 40 vs. others)	2.53	1.33-4.81	**0.005**
**IPSS risk** (Higher vs. Lower)	1.94	0.98-3.84	0.057
**AML transformation** (Yes vs. No)	3.58	1.53-8.35	**0.003**
**Prior to HSCT therapies** (Others vs. Appropriate treatment)	1.20	0.62-2.31	0.580
**Source of stem cell** (Not marrow vs. Marrow)	0.45	0.18-1.11	0.083
**Grade of aGVHD** (2-4 vs. Others)	4.01	1.54-10.41	**0.004**
**EBV infection** (Yes vs. No)	3.77	1.92-7.38	**< 0.001**
Cumulative incidence of non-relapse-mortality (NRM)			
**Age** (≥ 40 vs. Others)	1.63	1.09-2.45	**0.018**
**Blast** (≥ 5% vs.< 5%)	2.35	0.76-7.27	0.140
**WHO classification** (EB-1/EB-2 vs. Others)	0.29	0.09-0.56	**0.037**
**Prior to HSCT therapies** (Others vs. Appropriate treatment)	2.27	1.43-3.70	**< 0.001**
**Grade of aGVHD** (2-4 vs. Others)	2.96	1.93-4.55	**< 0.001**
**Grade of cGVHD** (Extensive vs. Others)	2.37	1.43-3.91	**< 0.001**

IPSS, International prognostic scoring system; IPSS-R, revised IPSS; DAC, decitabine; GVHD, graft-versus-host disease; aGVHD, acute GVHD; cGVHD, chronic GVHD; EBV, Epstein-Barr virus.

Significant p values (p < 0.05) are bolded.

## Discussion

To the best of our knowledge, this is the largest study to evaluate the use of HMAs (especially DAC), chemotherapy, and SC before transplantation in consecutive patients with MDS after long-term follow-up. An appropriate pre-transplant strategy (SC/HMA + <6 months) prolonged OS and decreased NRM in MDS patients receiving myeloablative allo-HSCT.

Disease status at the time of transplantation is one of the most important factors that influences outcomes after allo-HSCT (23), and the presence of more than 5% blasts at the time of transplantation is associated with poor prognosis (24). In our analysis, 24% of the HMA group and 42% of the chemotherapy group achieved CR at the time of HSCT (**Supplemental Table 1**). A similar result was observed in another clinical trial, with a higher haematological response to chemotherapy than AZA (25). We also assessed the impact of marrow blast on OS or RFS. The results were shown in **Supplemental Figure 6**. Overall, different blast levels did not impact OS (**sFigure 6A**, P=0.264). For RFS, patients with blast level of ≥15% showed a trend of worse RFS (**sFigure 6B**, P=0.056). Although chemotherapy was superior to HMA before transplantation, providing a better and more rapid reduction in the marrow blast percentage, it did not translate to a survival benefit, and the value of reducing the blast percentage before transplantation in MDS remains unclear (26). A recent study by Potter et al. using data from a large registry suggested that the post-transplant outcomes of patients with MDS who were not in CR were not significantly worse than those who were in CR (27). This is in line with our results that the marrow response was not an indepenW1dent predictor of a favourable prognosis for OS and RFS after transplantation. Furthermore, pre-transplant chemotherapy is associated with substantial morbidity and mortality, which may ultimately result in fewer patients undergoing HSCT. These findings suggest that achieving CR or mCR prior to HSCT is not a requisite for prolonged survival (27, 28). Taking the MAC conditioning into account, we prefer lower intensive regimes such as IAG or HAG instead of a 3 + 7 induction regimen for patients with higher-risk disease. In total, although the optimum strategy has not been determined, lower intensive regimes are commonly selected for higher-risk MDS patients with a transplant intention in our centre.

In our study, and in accordance with the report of Fenaux et al., treatment with HMA before transplantation prolonged OS and lowered the risk of relapse compared with treatment with conventional care in MDS (25). The mechanisms of AZA and DAC activity in MDS have been explored. HMA therapy may play a role in stabilizing the disease and may offer benefits with respect to relapse reduction *via* epigenetic modulation. Damaj et al. reported that 15% of AZA group patients experienced disease progression before transplantation compared with 51% of chemotherapy group patients (9). Similarly, 2% of patients in our study who received HMA experienced progression to AML before transplantation, which was significantly lower than the 15% of those who received chemotherapy. It has been reported that AZA can promote induction of CD8+ T-cell responses in AML (29). Administration of AZA post-transplantation increases the number of Tregs and induces a CD8+ T-cell response to a number of tumour antigens, including MAGE, BAGE, and Wilms’ tumour antigen 1 (29). Addition of DAC to conditioning therapy also induces tumour-associated antigen-specific T-cell responses, augmenting a greater graft-versus-leukaemia response after HSCT (30).

Although it is the only curative treatment available for patients with MDS, allo-HSCT is associated with a significant risk of early morbidity and mortality and therefore is not used as first-line treatment in MDS. For patients who are candidates for HSCT, choosing the optimal time to perform HSCT remains a major challenge in clinical practice. According to Markov model decision analyses, for patients with advanced IPSS risk (intermediate-2 and high), the strategy that maximizes discounted life expectancy is transplantation at the time of diagnosis (14). Similar to the results from decision analyses, our findings support that proceeding to HSCT for patients with higher IPSS-R risk within 6 months after diagnosis is associated with superior OS and RFS (**Supplemental Figure 4**). For those with lower IPSS risk (low and intermediate-1), watchful waiting and supportive care is appropriate, and delayed transplantation prior to the development of AML is the strategy that maximizes overall discounted life years (14). However, disease progression for lower risk patients is not always gradual and may be abrupt. Our results showed that early transplantation (within 6 months of diagnosis) may offer a survival advantage even in lower risk patients (**Supplemental Figure 3**). Considering that patients who did not undergo transplant were not included in our study, the conclusions should be treated with caution.

There are some limitations to this study. Next-generation sequencing (NGS) continues to identify specific mutations associated with disease progression. In general, transplant decision-making may be influenced by genetic changes identified using NGS, such as *TP53*, *RUNX1*, *ASXL1*, *RAS* and *JAK2* mutations (31, 32). Due to the limited data regarding NGS, we did not incorporate these data in our analysis. Most importantly, our single-centre study was retrospective, and therefore, the results need to be validated in multicentre clinical trials.

In conclusion, our study indicates that using an appropriate pre-transplant strategy (SC/HMA + <6 months) significantly prolongs OS and decreases NRM in MDS patients receiving myeloablative allo-HSCT. In the absence of randomized data, these results may help in clinical transplant decision-making for MDS patients.

## Data availability statement

The original contributions presented in the study are included in the article/**Supplementary Material**. Further inquiries can be directed to the corresponding authors.

## Ethics statement

The studies involving human participants were reviewed and approved by the Ethics Committee of the First Affiliated Hospital of Soochow University. Written informed consent to participate in this study was provided by the participants’ legal guardian/next of kin.

## Author contributions

HW, QW, JQ, XL, TC: contribution of patients, acquisition of data, analysis and interpretation of data. YH, DW and HW: study design, acquisition of funding, contribution of patients, interpretation of data, supervision of the study, and revision of the manuscript. HQ, CF, XT and CR: contribution of patients and revision of the manuscript. HW, QW and JQ wrote the paper. All authors read and approved the final manuscript.
