# Supplementary material for: Appropriate pre-transplant strategy for patients with myelodysplastic syndromes receiving allogeneic haematopoietic stem cell transplantation after myeloablative conditioning
Source: Front Immunol. 2023 Feb 28;14:1146619. doi: 10.3389/fimmu.2023.1146619 (PMC10011085; doi:10.3389/fimmu.2023.1146619)

**Supplemental Table1**

The impact of marrow blast at diagnosis on treatment response in MDS patients.

| Blast | No. | CR or mCR (%) |
| --- | --- | --- |
| <5% | 132 | 5.3 |
| 5%-9% | 120 | 25.8 |
| 10-14% | 71 | 39.4 |
| ≥15% | 48 | 45.9 |

**Supplemental Figure legends**

**Supplemental Figure 1** (**A**) Overall survival, (**B**) relapse-free survival, (**C**) cumulative incidence of relapse, and (**D**) cumulative incidence of non-relapse mortality (NRM) according to the prior-to-transplantation treatment received. SC, supportive care; HMA, hypomethylating agents (decitabine and azacitidine); Chemo, chemotherapy.

**Supplemental Figure 2** (**A**) Overall survival, (**B**) relapse-free survival, (**C**) cumulative incidence of relapse, and (**D**) cumulative incidence of non-relapse mortality (NRM) according to the different intervals between diagnosis and transplantation.

**Supplemental Figure 3** (**A**) Overall survival, (**B**) relapse-free survival, (**C**) cumulative incidence of relapse, and (**D**) non-relapse mortality (NRM) in lower risk MDS patients according to different intervals between diagnosis and transplantation.

**Supplemental Figure 4** (**A**) Overall survival, (**B**) relapse-free survival, (**C**) cumulative incidence of relapse, and (**D**) non-relapse mortality (NRM) in higher risk MDS patients according to different intervals between diagnosis and transplantation.

**Supplemental Figure 5** (**A**) Overall survival, (**B**) relapse-free survival, (**C**) cumulative incidence of relapse, and (**D**) non-relapse mortality (NRM) in MDS patients according to different pre-transplant strategies.

**Supplemental Figure 6** (**A**) Overall survival, (**B**) relapse-free survival according to blast level in total cohort.


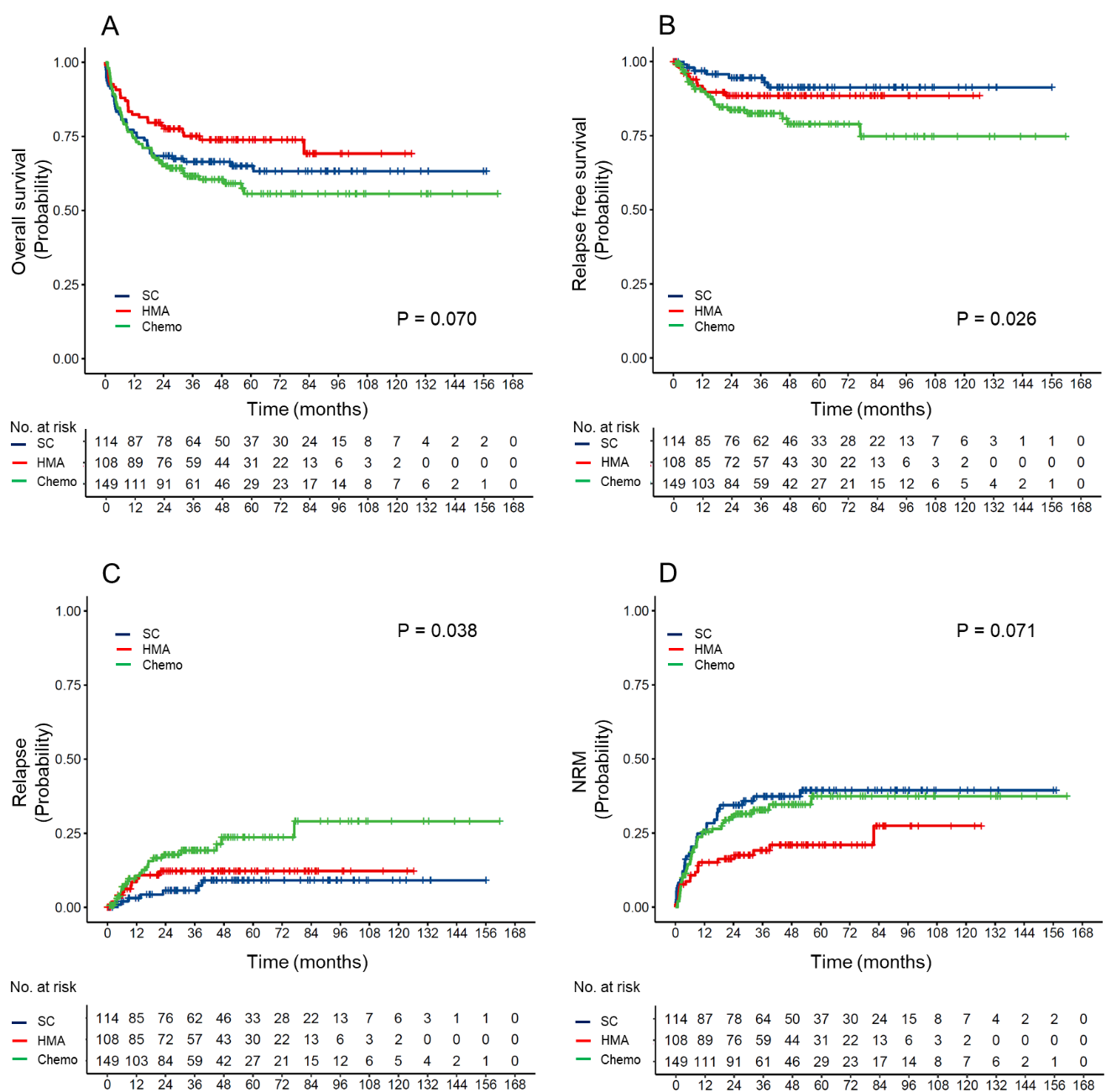
**Supplemental Figure 1**

**Supplemental Figure 2**


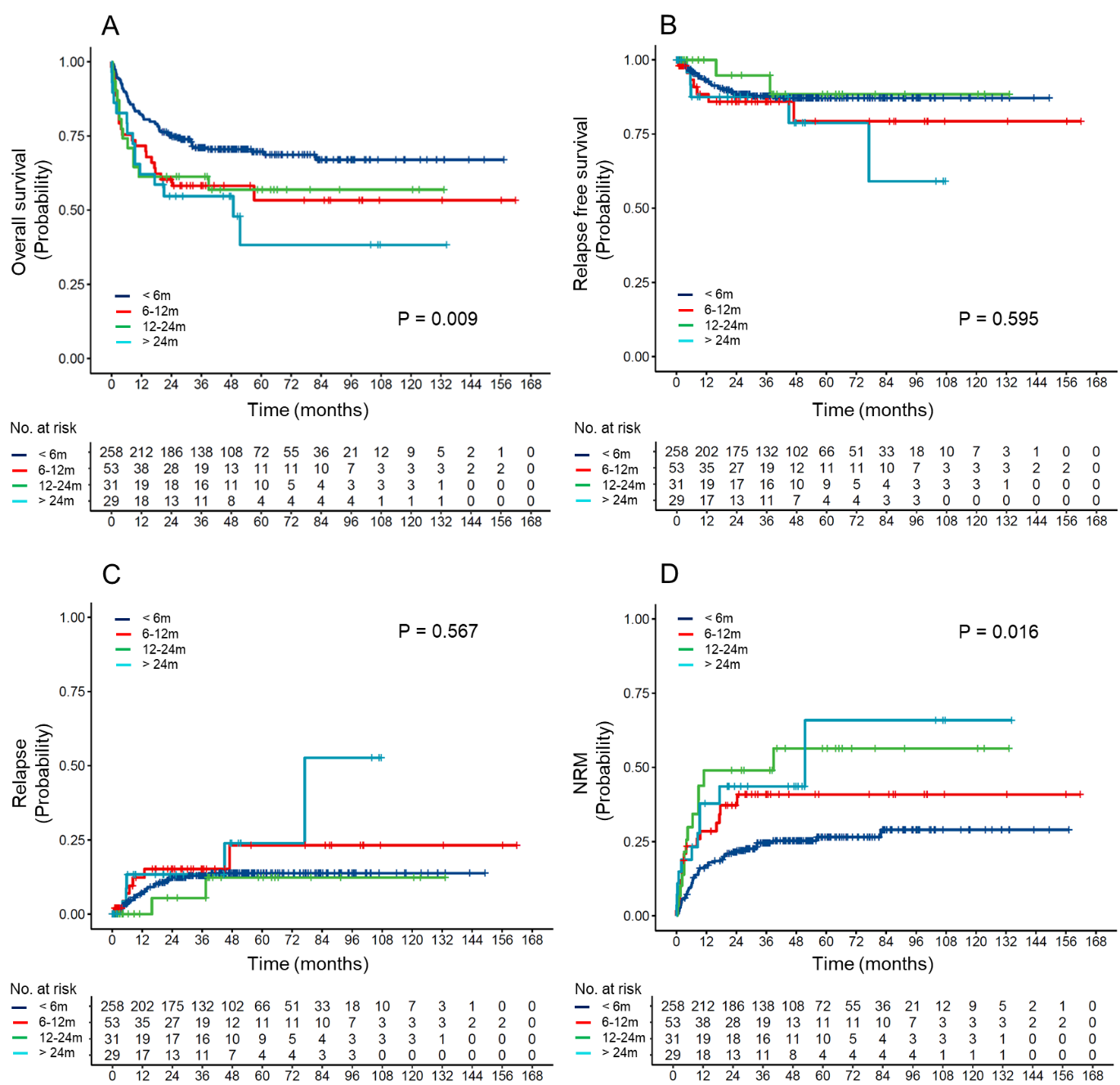


**Supplemental Figure 3**


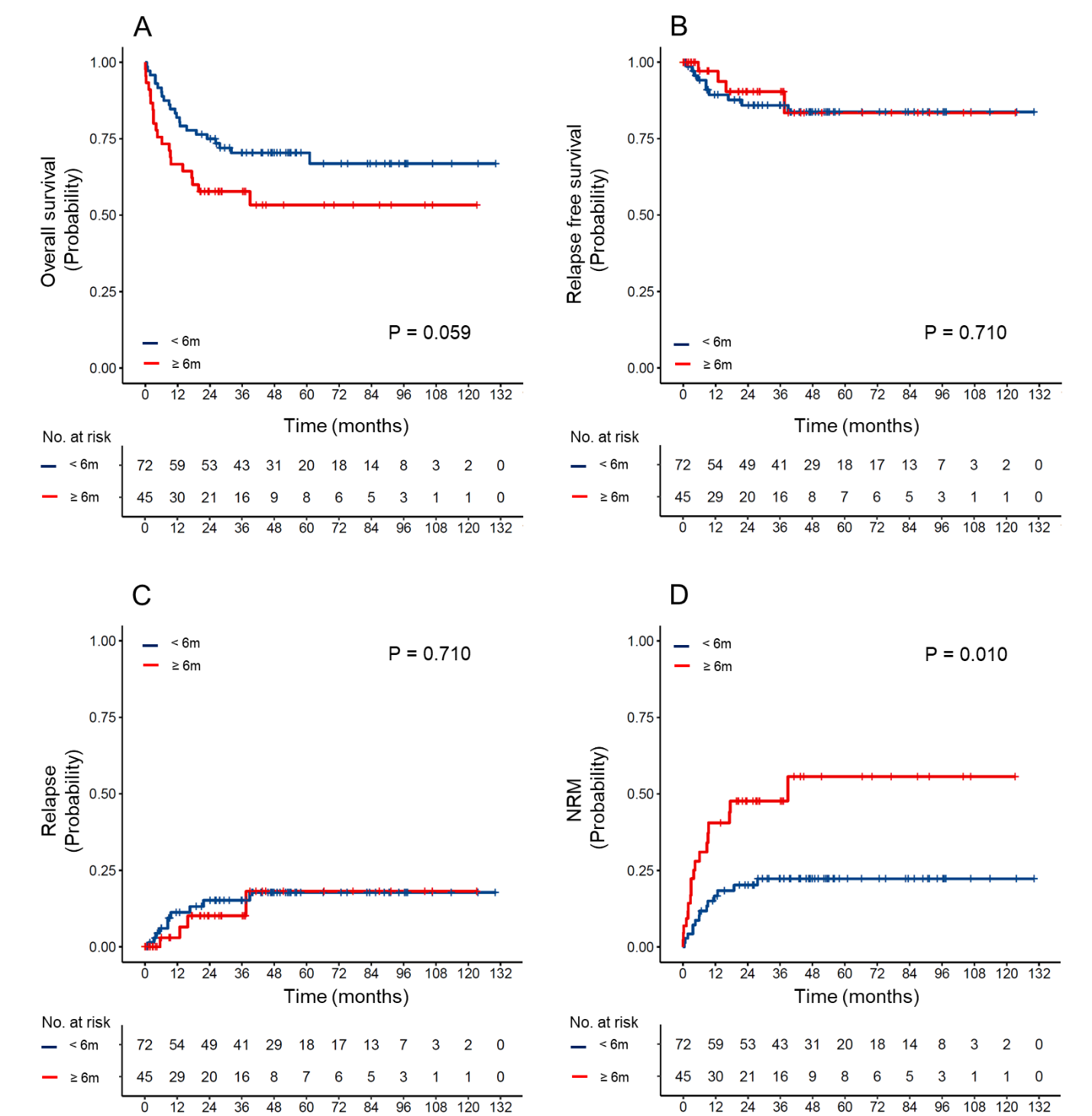


**Supplemental Figure 4**


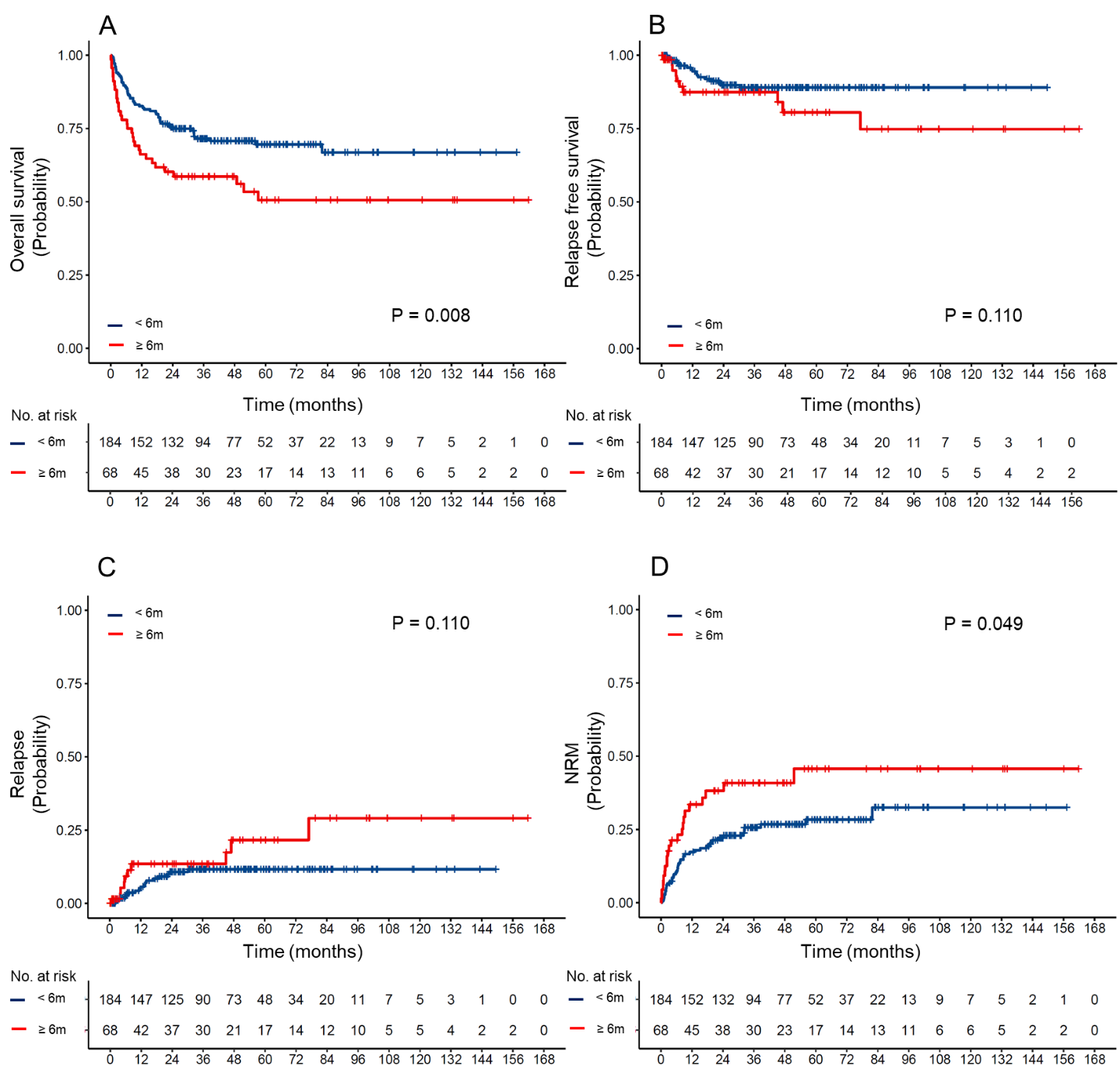


**Supplemental Figure 5**


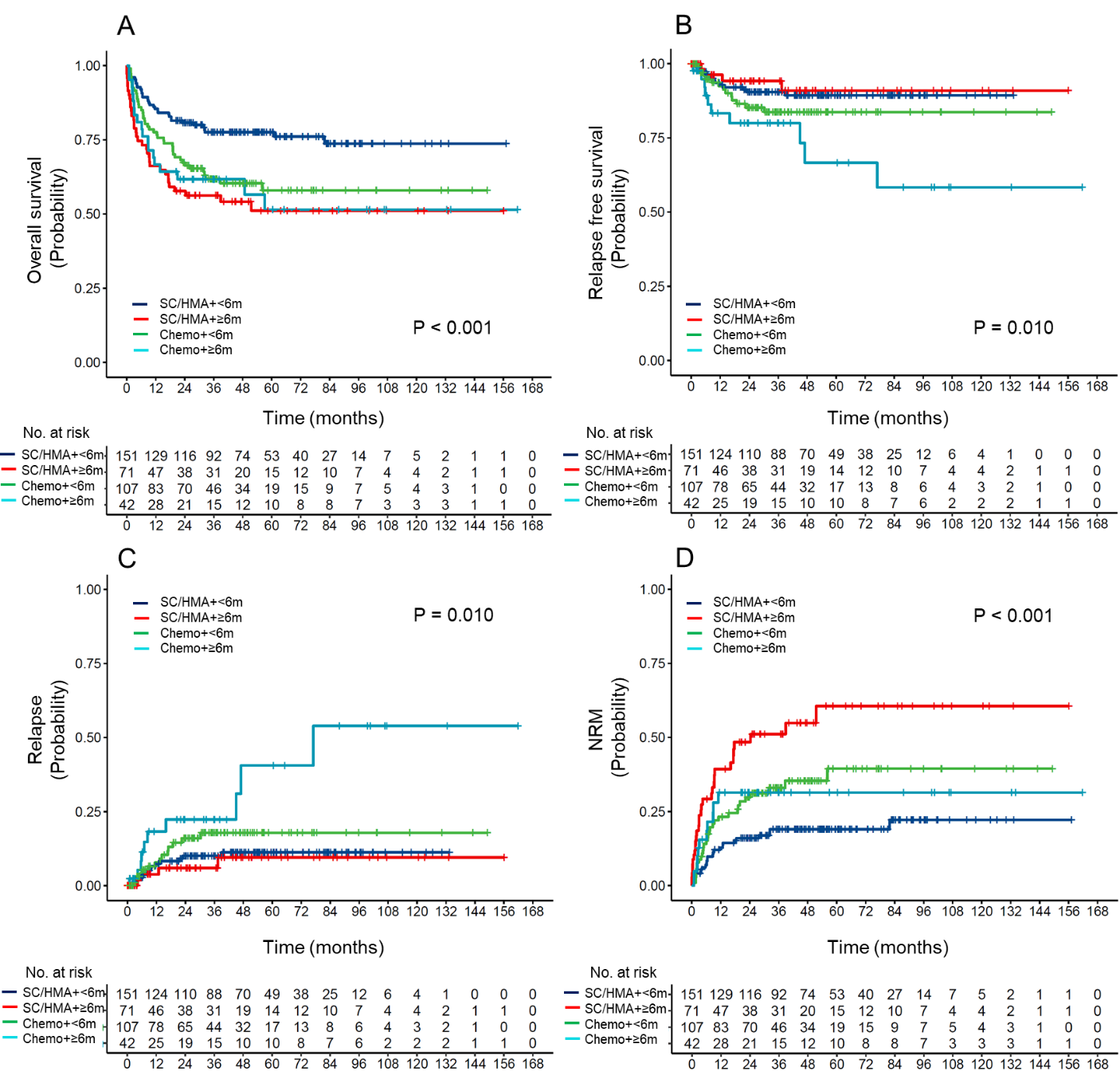


**Supplemental Figure 6**


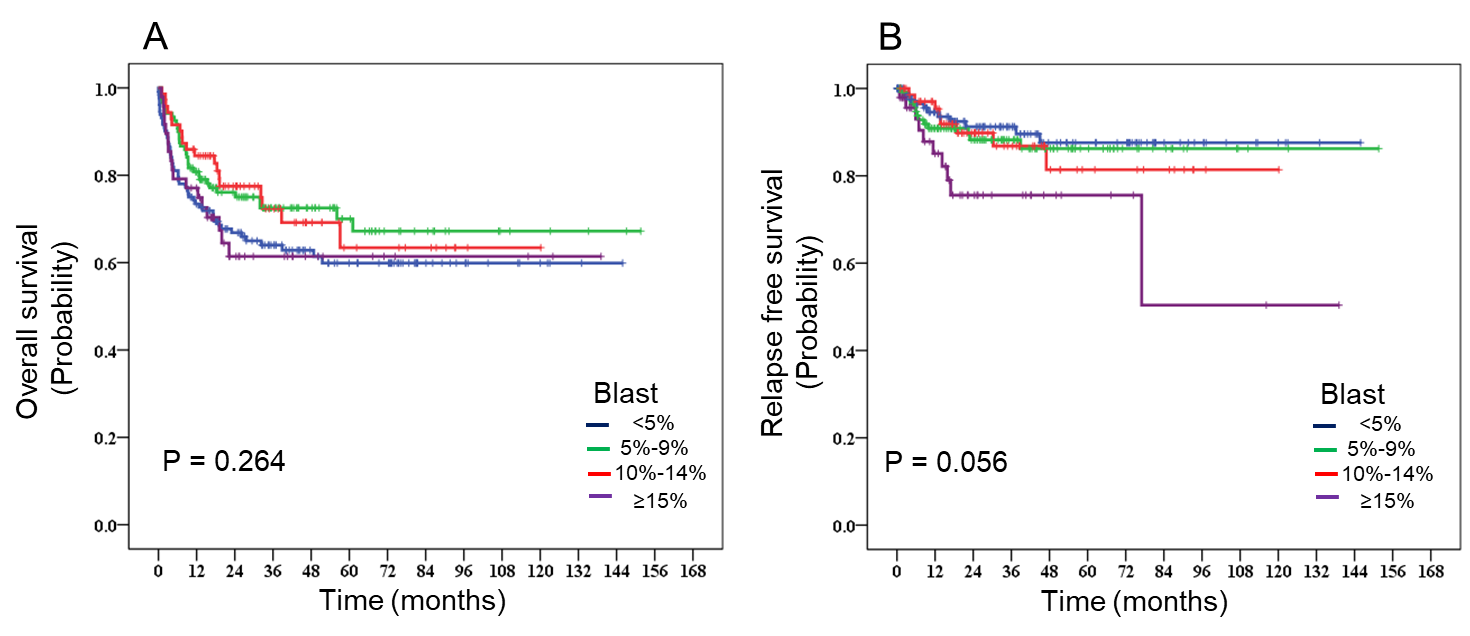

Supplement: Supplementary file 1 [file DataSheet_1.docx]
